# Reduced leukocyte mitochondrial copy number in metabolic syndrome and metabolically healthy obesity

**DOI:** 10.3389/fendo.2022.886957

**Published:** 2022-07-25

**Authors:** Rachel Agius, Nikolai Paul Pace, Stephen Fava

**Affiliations:** ^1^ Faculty of Medicine and Surgery, University of Malta, Msida, Malta; ^2^ Department of Medicine, Mater Dei Hospital, Msida, Malta; ^3^ Centre for Molecular Medicine and Biobanking, University of Malta, Msida, Malta

**Keywords:** mitochondrial DNA copy number, obesity, metabolic syndrome, insulin resistance, metabolic health

## Abstract

**Objective:**

This study aimed to investigate the associations between peripheral blood leukocyte mitochondrial copy number, metabolic syndrome, and adiposity-related body composition phenotypes in a high prevalence population.

**Methods:**

A single center cross-sectional study was conducted, consisting of 521 middle-aged subjects of Maltese-Caucasian ethnicity. Participants were stratified according to the presence of metabolic syndrome and different metabolic health definitions based on NCEP-ATP III criteria. Relative leukocyte mitochondrial DNA copy number was determined by quantitative polymerase chain reaction and corrected for leukocyte and platelet count. The associations between mitochondrial copy number and metabolic syndrome components was evaluated and adjusted for age and gender.

**Results:**

Significant negative correlations between mtDNA copy number and BMI, waist circumference, triglyceride levels, fasting plasma glucose, HbA1c, HOMA-IR and hsCRP were observed, along with a positive correlation with HDL-C levels. Mitochondrial copy number was lower in individuals with metabolic syndrome. When compared to metabolically healthy normal weight subjects, a reduction in mtDNA copy number was observed in both the metabolically healthy and unhealthy obese categories.

**Conclusion:**

Our data supports the association between reduced leukocyte mtDNA copy number, obesity, and metabolic syndrome. This investigation expands on the spectrum of associations between mtDNA copy number and metabolic phenotypes in different populations and underpins the role of mitochondrial dysfunction in the development and progression of metabolic syndrome and its components.

## Introduction

Obesity is typically accompanied by a cluster of multiple cardiometabolic abnormalities, including dysglycaemia, insulin resistance and dyslipidemia. These act as risk factors for the development of several obesity-related comorbidities, including type 2 diabetes mellitus (T2DM) and cardiovascular disease (1). The occurrence of cardiometabolic risk factors is not uniform in obesity. A unique subtype of obesity characterized by favorable metabolic profiles (termed metabolically healthy obesity) has been described (2). Conversely, a subset of lean individuals are metabolically abnormal (termed metabolically unhealthy normal weight) (3–5). Over the past few decades there has been an increased interest in characterizing the determinants and clinical outcomes of metabolic health. However, no unifying definition of what constitutes metabolic health exists to date. Most landmark studies use either the National Cholesterol Education Program – Adult Treatment Panel III (NCEP-ATPIII) definition of metabolic syndrome (or variations thereof), the presence of insulin resistance or a combination of the two (6–9). Recently, Zembic et al. derived an empirical definition of metabolic health based on cardiovascular and total mortality from the UK Biobank and NHANES-III datasets (10). Furthermore, the molecular mechanisms underlying the development of such body composition phenotypes remains largely unexplored. A growing body of evidence has implicated the important role of mitochondrial bioenergetics in metabolic disturbances, with several studies demonstrating that defects in mitochondrial function are associated with the metabolic syndrome, insulin resistance, obesity and T2DM (11–13).

Mitochondria are ubiquitous subcellular organelles with their own circular genome. They play a key role in cellular energy production and metabolic homeostasis by generating adenosine triphosphate (ATP) through oxidative phosphorylation. However, mitochondria are highly susceptible to oxidative stress as a consequence of unregulated reactive oxygen species (ROS) production, and the resultant dysfunction has been postulated to contribute towards the development of several chronic diseases (14, 15). The quantification of mtDNA copy number (mtDNA CN) is a surrogate index of cellular mitochondrial content. This has been increasingly employed as a biomarker of mitochondrial function that may reflect the degree of mtDNA damage (16). Mitochondrial dysfunction is associated with a decreased mtDNA CN and recent observations have shown that a reduction in mtDNA CN in several tissues (including leukocytes, skeletal myocytes, hepatocytes and white adipose tissue) correlates with visceral adiposity, BMI, hyperlipidemia, cardiovascular disease and mortality (17–19). Other studies have also demonstrated that chronic nutrient excess results in increased ROS production and altered mitochondrial morphology and function in tissues concerned with substrate metabolism such as adipocytes. Mitochondrial oxidative dysfunction leads to defective fatty acid oxidation and glucose homeostasis, resulting in abnormal lipid accumulation and insulin resistance. In turn, these factors contribute toward the pathogenesis of obesity and T2DM (20). More recently, several authors have provided additional evidence linking mtDNA CN with weight change. Meng et al. demonstrated a bi-directional and inverse relationship between mtDNA CN and weight gain in middle-aged females (21). Skuratovskaia et al. show that mtDNA levels increase within a year of bariatric surgery in obese subjects with T2DM (22). These findings highlight the dynamic relationship that exists between mtDNA levels and obesity but provide no direct insight into causal mechanisms.

While mtDNA depletion in hepatocytes and pancreatic beta cells leads to alterations in insulin secretion and glucose metabolism, the direct relationship between mtDNA CN and T2DM risk has yielded conflicting associations (23). Some studies have described a reduction in mtDNA content in both skeletal muscle and peripheral blood from T2DM subjects (24–26). Furthermore, depletion of mtDNA levels has also been postulated to precede the development of T2DM (27, 28). Directionally inconsistent associations between mtDNA content and glycemic parameters have also been described (29). Weng et al. demonstrated an increase in leukocyte mtDNA CN with a progressive deterioration in glucose metabolism (30). However, tissue-specific differences in mtDNA content in T2DM patients were reported, with a higher leukocyte but a lower muscle mtDNA content. This discrepancy has been attributed to the different tissue-specific effects of oxidative stress, driven by higher apoptosis and slower turnover of skeletal muscle cells (31). Other studies have also investigated the relationship between mtDNA CN and the metabolic syndrome, and show that a higher number of metabolic syndrome components correlates with a lower mtDNA CN (32, 33).

Although the association between leukocyte mtDNA CN depletion and metabolic syndrome is established, its correlation with healthy *vs* unhealthy subtypes of obesity has not been clearly evaluated. We hypothesized that individuals with unhealthy body composition phenotypes have a lower leukocyte mtDNA CN. In this study, we therefore sought to explore leukocyte mtDNA CN across different adiposity-related phenotypes using a middle-aged cohort from a high prevalence island population. We also evaluated which of the different definitions of metabolic health and their constituent components are associated with reduced leukocyte mtDNA CN.

## Materials and methods

### Ethics statement

This study was carried out in accordance with the Declaration of Helsinki and approved by the institutional ethics review board of the University of Malta (UREC MD 06/2016). Written informed consent was obtained from all participants.

### Subjects

The study population was comprised of 521 subjects of Maltese-Caucasian ethnicity. These were recruited through convenience sampling as part of an observational cross-sectional study aimed at characterizing body composition phenotypes in a Mediterranean island population having a high prevalence of obesity and cardiometabolic disease. Details of the research design and study protocol have been described elsewhere (34, 35). Briefly, a nationally representative sample of the non-institutionalized population aged 41 ± 5 years was recruited. Subjects with a history of type 1 diabetes, individuals with known underlying genetic or endocrine causes of overweight or underweight (apart from controlled thyroid disorders), individuals with a terminal illness or active malignancy, individuals who could not give their own voluntary informed consent and pregnant females were excluded. Baseline anthropometric, demographic, and clinical parameters (including lifestyle and medical comorbidities) were recorded at enrollment.

### Body size definitions

Body size phenotypes were generated based on the combined consideration of each participants’ BMI category and metabolic health. We used the following three definitions of metabolic health to cross-classify study participants:

a) Participants were considered to be metabolically healthy if they had one or none of the following NCEP-ATPIII components: high triglycerides (≥1.7 mmol/L) or lipid-lowering drugs, elevated systolic blood pressure (≥130 mmHg) or diastolic blood pressure (≥85 mmHg) or anti-hypertensive drugs, high fasting glucose (≥5.6 mmol/L) or medications for diabetes (insulin and oral anti-diabetic) and low HDL-C (<1.04 mmol/L for males and <1.29 mmol/L for females) (36). This definition does not incorporate waist circumference in view of its collinearity with BMI.

b) Absence of insulin resistance as defined by HOMA-IR <2.5. This cut-off value has been validated in other studies (37, 38).

c) An empirical definition of metabolic health based derived by Zembic et al. based on the risk of cardiovascular and total mortality from the NHANES-III and UK Biobank dataset (10). This classification defines metabolic health as follows: systolic blood pressure <130mmHg and no use of anti-hypertensive drugs, a WHR of <0.95 in females and <1.03 in males, and absence of diabetes.

Overweight and obese subjects were analyzed together as one aggregate category thus generating four body composition phenotypes: metabolically healthy normal weight (MHNW), metabolically unhealthy normal weight (MUHNW), metabolically healthy overweight or obese (MHOW/O) and metabolically unhealthy overweight or obese (MUHOW/O). Additionally, the presence or absence of metabolic syndrome (defined by ≥3 of the following NCEP-ATPIII parameters: waist circumference (WC) >102 cm in males and >88 cm in females; systolic/diastolic blood pressure (SBP/DBP) ≥130/85 mmHg or on antihypertensive medication; serum triglyceride level ≥1.69 mmol/l or on lipid-lowering medication; HDL-C <1.03 mmol/l in males and <1.29 mmol/l in females or on treatment aimed to increase HDL-C; fasting plasma glucose ≥5.6 mmol/l or on antihyperglycemic agents) was also applied.

### Biochemical analysis

Blood samples were taken from study subjects after an overnight fast. Hematologic and metabolic parameters (glycated hemoglobin, fasting plasma glucose (FPG), cholesterol fractions) were determined using standard hospital biochemical analyzers. Fasting insulin and high sensitivity CRP were quantified at baseline by sandwich ELISA (Diagnostic Automation, USA). Fasting insulin and FPG levels were used to determine the homoeostasis model assessment of insulin resistance (HOMA-IR) using the following formula: HOMA-IR = Fasting Insulin(μIU/mL) x Fasting Glucose (mmol/l)/22.5 (39).

### Determination of mtDNA copy number

Genomic DNA was extracted from peripheral blood leukocytes in EDTA anti‐coagulated blood using a QIAamp DNA extraction kit (Qiagen, Hilden, Germany). DNA quality and quantity were assessed using Nanodrop^®^ spectrophotometry (ThermoFisher Scientific), Qubit 3.0 (Life Technologies) and agarose gel electrophoresis. Extracted DNA was frozen at − 20°C for future use. Peripheral blood leukocytes were selected for mtDNA CN determination in view of their clinical utility as a minimally invasive disease biomarker and the mechanistic link between chronic inflammation and metabolic syndrome. Relative leukocyte mtDNA CN was determined by evaluating the ratio of mtDNA to nuclear DNA, using fluorescence-based quantitative polymerase chain reaction (qPCR). The mitochondrial gene *MT-CYB* encoding cytochrome b, and the nuclear gene encoding hemoglobin subunit β (*HBB*) as the single copy reference were used for relative quantification (26). Quantification was performed using a BioRad CFX96 Touch Deep Well Real-Time PCR Detection System^®^.

The primers complementary to the sequences of the *HBB* gene (forward: 5’-GAA GAG CCA AGG ACA GGT AC-3’; reverse: 5’-CAA CTT CAT CCA CGT TCA CC-3’) were used to amplify a 268-bp product as the nuclear single copy reference. The primer sequences complimentary to *MT-CYB* (forward: 5’-CCA ACA TCT CCG CAT GAT GAA AC-3’ and reverse 5’-TGA GTA GCC TCC TCA GAT TC-3’) amplified a 434bp amplicon. Reactions were carried out in optical 96-well plates, using 4µL of 5x HOT FIREpol^®^ EvaGreen ^®^ qPCR Mix (12.5mM MgCl_2_, dNTPs, EvaGreen^®^ dye and ROX dye – Solis BioDyne, Estonia), 20ng of genomic DNA, and 0.5µL of each of the forward and reverse oligonucleotides at 10µM concentration, and molecular biology grade water to make up a reaction volume of 20µL. qPCR amplification was performed under the following conditions: initial denaturation at 95°C for 300 seconds, followed by 30 cycles of denaturation at 95°C for 60 seconds, annealing at 56°C for 90 seconds and extension at 72°C for 120 minutes. Fluorescence signal acquisition was carried out in the extension phase. All assays were carried out in triplicate, and a no-template control was included in each run. Laboratory personnel were blinded with regards to case-control and disease status. Post-amplification melt curve analysis was performed to check for primer-dimer artifacts and to ensure reaction specificity. For melt curve analysis, the temperature was increased from 65 °C to 95 °C at 0.5 degree increments every 5 seconds.

Data collection was carried out using CFX Maestro ^®^ software. Baseline subtracted curve fitting with fluorescent drift correction was applied to generate a horizontal baseline. For each amplicon, melt curves (relative fluorescence units (RFU) per temperature for each well) and melt peaks (negative derivative of the RFU data per temperature for each sample) were displayed to confirm reaction specificity.

The ratio of mtDNA/nuclear DNA was calculated using the Pfaffl method, which is best suited for the interpretation of qPCR data where primer efficiency is not identical (40). Briefly, $\frac{E^{\text{ΔCt}CYTB}\;}{E^{\text{ΔCt}HBB}},$ where ΔCt is Ct*_HBB_* – Ct*_MT_*_-_ *_CYTB_* and *E* equals primer efficiency.

### Amplification specificity and PCR efficiency

To evaluate qPCR efficiency, standard curves were prepared by serial dilution of both *HBB* and *MT-CYTB* amplicons. The log_10_ of template copy number against the corresponding Ct value were plotted and linear regression analysis was applied (**Supplementary Figure 1**). For each reaction, PCR efficiency *E* was calculated using the standard curve points in the exponential phase using *E* = 10 ^-1/^ *^slope^*. The calibrator was a mixed DNA sample pooled from six randomly-selected normal weight metabolically healthy controls. The specificity of the amplification reaction was confirmed by melt curve analysis during temperature ramping, and by resolution of amplicons during agarose gel electrophoresis. **Supplementary Figure 2** shows melting curve (A) and melt peak (B) analysis of amplicons generated during qPCR. Both *MT-CYTB* and *HBB* amplicons demonstrated single melting peaks at 86.5 °C and 88 °C respectively. Electrophoretic separation of PCR products using agarose gels demonstrated prominent bands with the expected sizes (C). The standard curves for both *HBB* and *MT-CYTB* were linear over the serial dilution range (R^2^ = 0.99), and Cq values of unknown samples fell within the linear range. The gradient of the standard curves for *HBB* and *MT*-*CYTB* were -3.62 and -3.42 respectively, and the amplification efficiencies were 88.9% and 95.9% respectively. The inter-assay coefficient of variation for replicates in different batches was 2.1% and 1.8% for *HBB* and *MT-CYTB* respectively. The acceptable standard deviation for the triplicate threshold cycle (ΔCt) was set at 0.5, and runs were repeated for inacceptable SD values.

### Corrected mtDNA copy number

To control for possible contamination of leukocyte genomic DNA by mitochondrial DNA in platelet fractions from whole blood, a corrected leukocyte mtDNA CN was calculated as outlined by Hurtado-Roca et al. (41). Platelets contain only mtDNA and no nuclear DNA, and variation in platelet levels can confound relative mtDNA CN estimates from whole blood. A corrected count was calculated as follows: $mtDNACN_{leukocytes}=mtDNACN_{whole\;blood}=K\frac{Platelet\;count}{Leucocyte\;count}$ where *K* = 1.1.

### Statistics

Normality of continuous variables was assessed by the Shapiro-Wilk and Kolmogorov-Smirnoff tests. All continuous parameters exhibited a skewed non‐normal distribution, and non‐parametric statistics with medians and interquartile ranges are presented. Categorical variables are presented as percentages and the chi-square test was applied to compare dichotomous outcomes. To evaluate differences in quantitative variables between groups, the Kruskal–Wallis test was used for comparison between three or more categories, followed by Dunn’s *post hoc* test for pairwise comparison between subgroups. The independent samples Mann–Whitney U test was used for comparison between two categories. Spearman’s rank-order coefficient was used to explore the strength and direction of association between quantitative variables. The effect of mtDNA CN on metabolic syndrome was evaluated by binary logistic regression modelling adjusted for age, with mtDNA CN as the independent predictor and metabolic syndrome as the dependent response variable. The association between mtDNA CN and single components of the metabolic syndrome was also evaluated by binary logistic regression adjusted for age. In this analysis, each metabolic syndrome component was considered as a binary response variable, as the definitions include drug use for the management of hypertension, dyslipidemia and hyperglycemia. To account for the unequal representation of genders in the study population, regression analysis was additionally stratified by gender.

To further refine the association between adiposity, metabolic syndrome components and mtDNA CN, we applied principal components analysis (PCA) to reduce the dimensionality of the dataset. PCA was performed on nine standardized inter-correlated quantitative variables (WC, FPG HDL-C, TG, BMI, systolic BP, diastolic BP, HOMA-IR and hsCRP) using the FactoMineR package (42). A scree plot of eigenvalues was examined, and eigenvalues >1 were used to determine the number of selected factors. Orthogonal rotation (varimax) was taken to force variables strongly with a single component. Subsequently, the derived principal components were used as the dependent response variable in regression modelling, with mtDNA CN as the independent predictor and adjustment for age and sex.

Odds ratios and 95% confidence intervals are reported for a decrease in 10 mtDNA copies. Statistical analysis was performed using SPSS v26 and R v.3.4.2. A p value of <0.05 was considered statistically significant.

## Results

### Clinical and biochemical characteristics of the study cohort

The anthropometric, clinical, and biochemical characteristics of study cohort stratified according to the different classifications of metabolic health outlined above are summarized in **Table 1**. No significant difference in leukocyte count, platelet counts and in the proportion of leukocyte subpopulations was detected across body composition phenotypes or metabolic syndrome categories (data not shown).

**Table 1:** Baseline clinical and biochemical characteristics of the study cohort, stratified according to different metabolic health definitions.

	Metabolic health defined by 0 or 1 NCEP-ATPIII criteria				Metabolic health defined by Zembic et al				Metabolic health defined by HOMA-IR <2.5			
	MHNW (n=131)	MUHNW (n=25)	MHOW/OB (n=217)	MUHOW/OB (n=148)	MHNW (n=143)	MUHNW (n=13)	MHOW/OB (n=268)	MUHOW/OB (n=97)	MHNW (n=159)	MUHNW (n=7)	MHOW/OB (n=270)	MUHOW/OB (n=85)
Female (N/%)	109/83.2%	18/72%	144/66.3%	59/39.8%	116/81.1%	11/84.6%	161/60.1%	42/43.3%	118/74.2%	7/100%	154/57%	42/49.4%
Regular physical activity (N/%)	63/48.1%	14/56%	93/42.9%	53/35.8%	68/47.6%	9/69.2%	115/42.9%	31/32%	74/46.5%	2/28.6%	119/44.1%	26/30.6%
Smokers (N/%)	32/24.4%	6/24%	48/22.1%	31/20.9%	35/24.5%	3/23.1%	54/20.1%	25/25.8%	34/21.3%	3/42.9%	55/20.4%	22/25.9%
	**Median (IQR)**	**Median (IQR)**	**Median (IQR)**	**Median (IQR)**	**Median (IQR)**	**Median (IQR)**	**Median (IQR)**	**Median (IQR)**	**Median (IQR)**	**Median (IQR)**	**Median (IQR)**	**Median (IQR)**
Age (years)	41 (7)	41 (4)	40 (6)	42 (5)	41 (7)	39 (5)	41 (5)	42 (6)	41 (7)	39 (5)	41 (6)	42 (6)
BMI (kg/m2)	22.5 (2.5)	21.8 (3.3)	29 (6.1)	31.1 (7)	22.4 (2.8)	23.5 (2.5)	28.9 (5.9)	32.3 (7.2)	22.4 (2.7)	24 (1.8)	29.1 (5.7)	32.7 (7.1)
Systolic BP (mmHg)	115 (15)	125 (10)	120 (10)	122 (14)	118 (15)	125 (15)	120 (10)	125 (10)	120 (15)	120 (20)	120 (10)	120 (15)
Diastolic BP (mmHg)	80 (10)	80 (0)	80 (10)	80 (5)	80 (10)	80 (0)	80 (10)	80 (5)	80 (10)	80 (10)	80 (5)	80 (5)
Waist: hip ratio	0.82 (0.1)	0.85 (0.1)	0.89 (0.11)	0.93 (0.1)	0.83 (0.1)	0.85 (0.1)	0.89 (0.1)	0.97 (0.12)	0.82 (0.1)	0.85 (0.1)	0.9 (0.1)	0.93 (0.1)
TC (mmol/l)	4.58 (1.2)	5.08 (1.13)	4.79 (0.89)	5.1 (1.53)	4.63 (1.2)	5.08 (1.1)	4.89 (1.01)	4.9 (1.39)	4.64 (1.2)	5.71 (1.7)	4.86 (1.04)	5.09 (1.29)
LDL-C (mmol/l)	2.6 (0.98)	3.2 (1.06)	2.84 (0.94)	3.15 (1.3)	2.66 (1.2)	3.27 (1.4)	2.91 (1)	3.03 (1.2)	2.62 (1.1)	3.49 (1.2)	2.88 (1.01)	3.14 (1.2)
HDL-C (mmol/l)	1.66 (0.52)	1.5 (0.58)	1.41 (0.46)	1.27 (0.38)	1.63 (0.5)	1.82 (0.6)	1.4 (0.4)	1.16 (0.3)	1.63 (0.5)	1.29 (0.7)	1.39 (0.4)	1.09 (0.3)
TG (mmol/l)	0.75 (0.4)	0.86 (0.51)	0.97 (0.59)	1.53 (0.97)	0.77 (0.4)	0.76 (0.7)	1.07 (0.6)	1.44 (0.9)	0.77 (0.4)	0.58 (1.1)	1.07 (0.6)	1.7 (1.11)
FPG (mmol/l)	4.92 (0.5)	5.24 (1.1)	5.06 (0.5)	5.69 (0.8)	4.94 (0.6)	4.92 (0.4)	5.17 (0.6)	5.45 (1.3)	4.94 (0.6)	4.87 (0.5)	5.13 (0.6)	5.7 (1.61)
HbA1c (%)	5.2 (0.4)	5.2 (0.2)	5.2 (0.4)	5.5 (0.55)	5.2 (0.3)	5.2 (0.29)	5.3 (0.4)	5.6 (1.3)	5.2 (0.3)	5.1 (0.6)	5.3 (0.49)	5.6 (1.3)
UA (mmol/l)	242 (83)	255 (103)	280 (85)	315 (123)	242 (91)	255 (77)	289 (92)	314 (123)	242 (91)	276 (96)	290 (96)	307 (106)
HOMA-IR	1.13 (0.9)	1.13 (0.96)	1.69 (1.1)	2.2 (1.3)	1.13 (0.9)	1.39 (1)	1.75 (0.9)	2.44 (1.5)	1.12 (0.8)	2.67 (0.2)	1.64 (0.8)	3.09 (0.9)
hs CRP (mg/l)	3.5 (4)	4.2 (3.6)	4.6 (4.4)	5.1 (4.6)	3.5 (3.8)	6.1 (4.1)	4.7 (4.5)	5.15 (4.3)	3.6 (3.9)	4.2 (4.2)	4.6 (4.3)	6.2 (4.1)

IQR, interquartile range; NCEP-ATPIII, National Cholesterol Education Program – Adult Treatment Panel III; MHNW, Metabolically healthy normal weight; MUHNW, Metabolically unhealthy normal weight; MHOW/O, Metabolically healthy overweight/obese; MUHOW/O, Metabolically unhealthy overweight/obese; FPG, Fasting plasma glucose; TC, Total Cholesterol; BMI, Body mass index; BP, Blood pressure; LDL-C, Low density lipoprotein -cholesterol; HDL-C, High density lipoprotein -cholesterol; TG, Triglycerides; UA, Uric acid.

### mtDNA copy number and metabolic indices

**Figure 1:**
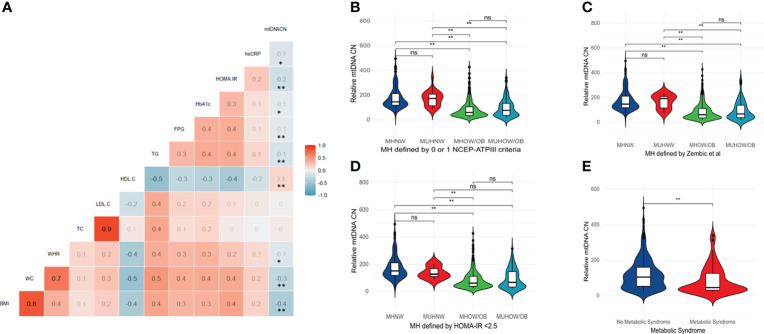
**(A)** Correlation matrix between relative mtDNA copy number and key metabolic parameters – BMI, WC, WHR, TC, LDL-C, HDL-C, TG, FPG, HbA1c, HOMA-IR and hsCRP. Color scale depicts Spearman’s rank-order correlation coefficient. ** Correlation is significant at the 0.01 level (2-tailed). * Correlation is significant at the 0.05 level (2-tailed). **(B–E)** Violin plots depicting mtDNA CN differences between body composition phenotypes and across different definitions of metabolic health and the metabolic syndrome. A significantly lower mtDNA copy number was observed in individuals with metabolic syndrome (Mann-Whitney U test, p < 0.05). A significantly lower copy number was present in both the MHOW/O and MUHOW/O categories, compared to MHNW participants (Kruskal-Wallis test, p <0.001), across different metabolic health definitions. The violin plots reflect data distribution. The centerline in the box plot illustrates the medians; box limits indicate the 25th and 75th percentiles; whiskers extend 1.5 times the interquartile range from the 25th and 75th percentiles. ** significant difference at p < 0.01. NS, not significant; MHNW, Metabolically healthy normal weight; MUHNW, Metabolically unhealthy normal weight; MHOW/O, Metabolically healthy overweight/obese; MUHOW/O, Metabolically unhealthy overweight/obese; CN, Copy Number; NCEP-ATPIII, National Cholesterol Education Program – Adult Treatment Panel III; FPG, Fasting plasma glucose; TC, Total Cholesterol; BMI, Body mass index; BP, Blood pressure; LDL-C, Low density lipoprotein -cholesterol; HDL-C, High density lipoprotein -cholesterol; TG, Triglycerides; UA, Uric acid; WHR, Waist: Hip Ratio.

The relationship between relative mtDNA CN and metabolic parameters was investigated by Spearman’s correlation. A correlation matrix of mtDNA CN with quantitative metabolic parameters is provided in **Figure 1A**. Significant negative correlations between mtDNA CN and BMI, waist circumference, waist: hip ratio, triglyceride levels, fasting plasma glucose, HbA1c, HOMA-IR and hsCRP were observed, along with a positive correlation with HDL-C levels. No significant correlation with age was observed (r_s_=0.03, p =0.497). No difference in mtDNA CN between genders was detected (Mann-Whitney U test, p = 0.065)

We next explored the relationship between mtDNA CN and the number of NCEP-ATPIII components that define the metabolic syndrome. A significant decrease in median mtDNA CN with an increase in metabolic syndrome components was observed (Kruskal-Wallis test, p <0.001 – **Supplementary Figure 3**. A significantly lower mtDNA CN was similarly observed in individuals with metabolic syndrome (Mann-Whitney U test, p < 0.001) – **Figure 1E**. When the study cohort was categorized according to different metabolic health definitions (metabolic health defined by 0 or 1 of the NCEP-ATPIII criteria, metabolic health defined by HOMA-IR < 2.5 and metabolic health according to the empirical definition by Zembic et al), a significantly lower median mtDNA CN was present in both the MHOW/O and MUHOW/O categories, compared to MHNW participants (Kruskal-Wallis test, p <0.001) (**Figures 1B**–**D**). No significant difference in relative mtDNA CN between the MHOW/O and MUHOW/O was detected. No significant differences in mtDNA CN were detected between MHNW and MUHNW across different definitions, although the small number of subjects in the MUHNW category restricts this comparison. **Table 2** summarizes mtDNA CN values across definitions of metabolic health based on different cross-classifications, with pair-wise comparisons between categories.

**Table 2:** Relative mtDNA copy number across definitions of metabolic health based on 0 or 1 NCEP-ATPIII criteria, HOMA-IR < 2.5, the empirical definition by Zempic et al. and the metabolic syndrome.

Metabolic health definition		Relative mtDNA copy number										
		n	Median	Q75	Q25	p value^a^	p value^b^	p value^c^	p value^d^	p value ^e^	p value ^f^	p value ^g^
**Metabolic health defined by 0 or 1 NCEP-ATPIII parameters**	MHNW	131	145.5	211.1	117.7	<0.01	<0.01	NS	NS	<0.01	<0.01	–
	MUHNW	25	172.3	207.0	114.0							
	MHOW/O	217	59.2	105.2	34.9							
	MUHOW/O	148	76.0	133.0	35.4							
**Metabolic health defined by HOMA-IR < 2.5**	MHNW	147	152.1	212.7	118.9	<0.01	<0.01	NS	NS	NS	0.018	–
	MUHNW	7	125.0	199.6	107.4							
	MHOW/O	270	59.9	110.2	36.4							
	MUHOW/O	85	65.3	143.5	31.5							
**Metabolic health defined by Zembic et al**	MHNW	143	146.1	211.1	117.7	<0.01	<0.01	NS	NS	<0.01	<0.01	–
	MUHNW	13	191.3	207.0	115.1							
	MHOW/O	268	60.4	111.5	34.9							
	MUHOW/O	97	67.2	137.3	37.1							
**Metabolic Syndrome**	Absent	433	105.2	160.1	53.2	–	–	–	–	–	–	<0.01
	Present	88	46.2	126.4	31.0							

p value^a^ MHNW vs MUHOW/O.

p value^b^ MHNW vs MHOW/O.

p value^c^ MHOW/O vs MUHOW/O.

p value^d^ MHNW vs MUHNW.

p value^e^ MUHNW vs MUHOW/O.

p value^f^ MUHNW vs MHOW/O.

p value^g^ Metabolic syndrome vs No metabolic syndrome.

MHNW, Metabolically healthy normal weight; MUHNW, Metabolically unhealthy normal weight; MHOW/O, Metabolically healthy overweight/obese; MUHOW/O, Metabolically unhealthy overweight/obese; Q75, Upper quartile; Q25, lower quartile; NCEP-ATPIII, National Cholesterol Education Program – Adult Treatment Panel III; NS, not significant.

Logistic regression models were applied to evaluate the association between mtDNA CN and 1) metabolic syndrome 2) each of the NCEP-ATPIII components as outcome variables 3) the metabolically unhealthy state [based on the empirical definition by Zembic et al] and 4) HOMA-IR ≥2.5. In the age adjusted models, a 10-fold reduction in mtDNA CN was associated with a marginally higher odds of the metabolic syndrome in both genders, increased triglycerides in males, and increased waist circumference in females only. A reduction in mtDNA CN was associated with a minor but significantly higher odds of both the metabolically unhealthy phenotype in both genders and HOMA-IR ≥ 2.5 in females. Results of regression models in the overall cohort and stratified by gender are presented in **Table 3**.

**Table 3:** Results of age adjusted binary logistic regression analysis between mtDNA copy number and metabolic syndrome, its individual components, the metabolically unhealthy phenotype [based on the empirical definition by Zembic *et al*] and HOMA-IR ≥ 2.5.

	Overall			Males			Females		
Dependent variable	OR	95%CI	p-value	OR	95%CI	p-value	OR	95%CI	p-value
**Metabolic syndrome**	1.05	1.02-1.09	**0.002**	1.04	1.01-1.09	**0.040**	1.06	1.01-1.12	**0.011**
**Increased triglycerides**	1.04	1.01-1.08	**0.015**	1.02	1.00-1.05	**0.010**	1.05	0.99-1.11	0.075
**Increased FPG**	1.02	1.00-1.05	0.094	1.02	0.98-1.06	0.258	1.02	0.98-1.06	0.287
**Hypertension**	1.02	1.00-1.04	0.102	1.03	0.99-1.07	0.111	1.01	0.99-1.04	0.113
**Increased waist circumference males**	-			1.01	0.97-1.05	0.561	-		
**Increased waist circumference females**	-			-			1.07	1.04-1.11	**0.010**
**Reduced HDL-C males**	-			1.03	0.98-1.08	0.257	-		
**Reduced HDL-C females**	-			-			1.03	0.99-1.06	0.184
**Metabolically unhealthy phenotype**	1.04	1.02-1.09	**0.039**	1.07	1.03-1.12	**0.03**	1.04	1.02-1.20	**0.04**
**HOMA-IR ≥2.5**	1.03	1.01-1.07	**0.029**	1.01	0.97-1.06	0.060	1.06	1.01-1.10	**0.016**

Odds ratios are given for a ten-fold reduction in mtDNA copy number in the overall cohort and stratified by gender.

### Principal components analysis

**Figure 2:**
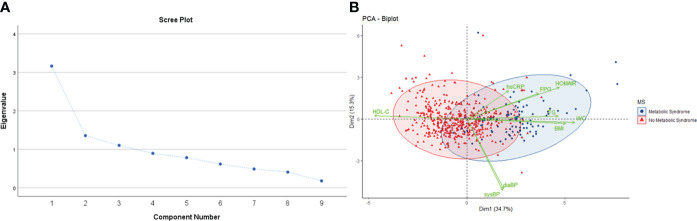
**(A)** Scree plot showing eigenvalue against all factors. Three factors have eigenvalues >1, and collectively explain 62.5% of the cumulative variance in the dataset. **(B)** PCA biplot shows individual observations as datapoints, colored according to presence or absence of metabolic syndrome. Points are plotted on a plane formed by the first two principal components. The original variables are shown as green vectors from the origin. The orientation of the vector with respect to the principal component space represents its contribution to the PC.

Since different adiposity/metabolic parameters are interrelated and converge physiologically to determine the causal trajectory to cardiometabolic disease, we used PCA to reduce the dimensionality of the dataset and explore its relationship with mtDNA CN. PCA with orthogonal (varimax) rotation was conducted on 9 items as outlined earlier. Bartlett’s test of sphericity indicated that the correlations were sufficiently large for a PCA (χ2 = 1299, p <0.01). An initial analysis was run to obtain eigenvalues for each data component. Three principal components (PC) had eigenvalues > 1 and in combination explained 62.5% of data, and a scree plot justified retaining 3 factors in the final analysis (**Figure 2**). The rotated component matrix showed that PC1 has high loading for WC and BMI, PC2 for blood pressure and PC3 has high loading for FPG, HOMA-IR and TG (**Supplementary Table 1**).

The three PC were incorporated as response variables in regression models to test their association with mtDNA CN as the predictor. After adjustment for age and gender, an inverse association between PC1 (adiposity parameters), PC3 (insulin resistance) and mtDNA CN was detected. The other PC, defined by blood pressure, showed no significant association with mtDNA CN (**Table 4**). This analysis supports the association of excess adiposity and insulin resistance with reduced relative mtDNA CN obtained from univariate analysis.

**Table 4:** Regression estimates for each of the three PC derived from PCA, representing adiposity (PC1) insulin resistance (PC3) and hypertension (PC2).

Dependent variable	β	95%CI	p value
**PC1 (adiposity)**	-0.03	(-0.04) - (-0.02)	**<0.001**
**PC3 (insulin resistance)**	-0.01	(-0.02) - (0.001)	**0.016**
**PC2 (hypertension)**	-0.006	(-0.01) - (0.01)	0.904

PC1 and PC3 showed a strong significant inverse association with mtDNA CN. Regression coefficient β is reported for an increase in 10 mtDNA copies.

## Discussion

In this cross-sectional study we explore the association between peripheral blood mtDNA CN and the odds of metabolic syndrome and its constituent components. After adjustment for both clinical covariates and blood cell composition as potential confounders, we demonstrate a minimal but significant inverse association between the two parameters. Our findings are relevant and new to a regional Southern European island population that bears a high burden of obesity, type 2 diabetes and cardiometabolic disease. We also demonstrate that a reduced mtDNA CN is present in both healthy vs unhealthy subtypes of obesity relative to the healthy normal-weight category. Thus, mtDNA CN appears to hold limited utility as a biomarker in stratifying the two subtypes of obesity. To the best of our knowledge, this is the first study that directly evaluates associations between mtDNA CN and metabolically healthy *vs* unhealthy subtypes of obesity as outcomes.

This manuscript provides additional perspective into obesity-associated mitochondrial dysfunction and reinforces the challenges of risk stratification of this complex trait. The concept of metabolically ‘benign’ obesity with preservation of insulin sensitivity is established, with some studies identifying a favorable cardiometabolic profile with reduced incidence of type 2 diabetes compared to unhealthy obesity (43, 44). This is reinforced by evidence from large prospective cohort studies showing that MHO does not carry an increased risk of cardiovascular or total mortality (10). Notwithstanding, this model is not without its controversies, and a strong unresolved debate concerns whether ‘obese but healthy’ individuals are truly free of adverse cardiovascular outcomes (45). This investigation showed that the difference in relative mtDNA CN between the MHOW/O and MUHOW/O did not exceed statistical significance thresholds, suggesting that obesity parameters are the strongest associates of reduced mtDNA CN in the study cohort. This finding is broadly congruent with evidence from metaanalysis showing that even in the absence of metabolic abnormalities, obese individuals are at increased risk of adverse long-term outcomes (46). Clinically, the observed associations further underscore the importance of regulating body weight even in the absence of the metabolic syndrome. In the broader context, it is important to emphasize that the NCEP-ATPIII metabolic parameters exhibit collinearity due to overlap of physiological mechanisms, and thus they should not be considered as mutually exclusive traits. Rather, they represent surrogate indices of insulin resistance. Stratifying obesity phenotypes based on the number of NCEP-ATPIII components assumes arbitrarily that the risk factors are metabolically equivalent. This generalization may be inaccurate, as even mild elevations in plasma glucose lead to secondary effects *via* alterations in lipid metabolism and generate reactive oxygen species and proinflammatory cytokines (47, 48). The notion of metabolic health as a static concept may also underestimate the long-term adverse effects of weight gain from baseline, and it does not provide any insight into obesity-related comorbidity and its prognosis (49, 50). The binary characterization of obesity into metabolically healthy *vs* unhealthy subtypes bypasses important considerations. These include cardiorespiratory fitness, which has been shown to explain both all-cause and cardiovascular mortality in obesity (51). Body composition phenotypes based on anthropometric indices do not capture functional differences between subcutaneous and visceral fat, which require imaging-based assessment (52, 53). Furthermore, differences in fat distribution patterns underlie variation in cardiometabolic disease risk across the BMI continuum. Adipose tissue distribution in the lower subcutaneous (gluteo-femoral) region is associated with a lower cardiometabolic risk, independent of precisely measured markers of visceral fat mass particularly in normal weight individuals (54, 55). In meta-regression analysis both waist circumference and the waist-to-hip ratio were associated with elevated incident CVD risk in both sexes (56). Recently, the Brazilian Longitudinal Study of Adult Health reported that a higher lower limb: trunk fat ratio was related to a reduced 10-year cardiovascular risk, mediated by lower systolic blood pressure, lower total cholesterol, and increased HDL-C levels (57, 58). Central fat depots contain dysfunctional adipocytes characterized by a pro-inflammatory nature, restricted hyperplasia, and higher turnover of bioactive lipids. Conversely, lower (gluteo-femoral) fat depots have the capacity to expand by recruiting additional adipocytes during periods of positive energy balance to accommodate fat undergoing redistribution, thereby protecting against ectopic fat deposition. Gluteo-femoral fat depots are also associated with a lower lipid turnover and are less pro-inflammatory, thereby being less detrimental to metabolic health (59, 60). These findings imply that categorizing body phenotypes into either metabolically healthy or unhealthy obese can create a false dichotomy, since both obesity and cardiometabolic disease are dynamic states along a pathophysiological continuum.

Broadly, the findings from this study are consistent in direction and magnitude with other investigations that report comparable associations between reduced mtDNA CN and several chronic diseases, including cardiometabolic outcomes (61, 62). The association between reduced mtDNA CN and obesity, diabetes, dyslipidemia and hypertension has been demonstrated by robust cross-ancestry analysis of large cohorts from TOPMed and UK Biobank (63). Other smaller-scale studies have similarly demonstrated depletion of leukocyte mtDNA CN in metabolic syndrome (32, 33). Our findings thus merit critical interpretation in the context of the study cohort characteristics. The study population described here comprises a carefully phenotyped homogenous cohort of middle-aged adult subjects. This contrasts with the older and broader age range reported in literature on mtDNA CN and metabolic syndrome. Importantly, within a middle-aged population, the likelihood of survival bias causing underestimation of effect size is less likely. Additionally, sarcopenic obesity, defined by the age-related decline in muscle mass coupled with higher adiposity and insulin resistance is uncommon in this age group (64, 65). mtDNA levels also decline with chronological age (66). These factors underscore the clinical relevance of utilizing the selected age group to evaluate associations between mtDNA CN and metabolic outcomes.

This investigation thus expands on the spectrum of established associations between mtDNA CN and metabolic phenotypes in different populations. The population genetic element is highly relevant in the context of mtDNA CN, as recent genome wide association scans have identified several independent loci that regulate mtDNA CN (67, 68). In addition, mitochondrial haplogroups have been associated with incident obesity in different populations, and mtDNA CN has been shown to correlate with environmental factors such as fine particulate matter (PM_2.5_) and components of the built environment (69–71). The Maltese population has an alarming obesity problem that is compounded by an ‘obesogenic’ environment and a limited infrastructure for active living (72). It is thus essential for molecular epidemiological studies to consider the impact of external factors on mtDNA CN and disease risk.

Deriving meaningful comparisons to identical studies is challenging, particularly in view of heterogeneity in patient ascertainment criteria, differences in mtDNA CN determination techniques (qPCR *vs* digital PCR *vs* high throughput sequencing), a lack of a uniform definition of metabolic health, variation in background prevalence of obesity and differences in study design (cross-sectional *vs* longitudinal). Additionally, cohort-specific aspects, including sociodemographic factors such as education level, physical activity and economic status impact on the risk and progression of metabolic outcomes and can potentially represent hidden confounders.

Notwithstanding these factors, mtDNA CN holds considerable interest as a minimally invasive biomarker. Importantly, it is physiologically relevant in the context of cellular metabolism and regulation of gene expression. Changes in mitochondrial content determine nuclear gene expression patterns through retrograde signaling, and Guantes et al. showed a direct effect on mRNA abundance, translation, and alternative splicing (73). mtDNA CN also regulates nuclear expression *via* changes in CpG methylation (74). Jeng et al. demonstrate that *in-vitro* depletion of mtDNA reduces mitochondrial proteins involved in oxidative phosphorylation (75). There is also robust evidence causally linking changes in mtDNA CN to adipose tissue inflammation and oxidative stress. Changes in mitochondrial respiratory capacity can impact on macrophage polarization and the resulting chronic subclinical inflammation that is associated with both metabolic syndrome and atherosclerotic processes. Proinflammatory M1 macrophages generate ATP primarily through glycolysis, while anti-inflammatory M2 macrophages rely on oxidative phosphorylation (OX-PHOS) and mitochondrial electron transport (76). Plausibly, a reduced mtDNA CN results in insufficient OX-PHOS proteins and a block in the reprogramming of M1 macrophages to the M2 subtype (62). Adipose tissue macrophages (ATMs) infiltrate adipose tissue in obesity, and M1-activated cells secrete an array of proinflammatory cytokines that drive insulin resistance, while M2-cells promote insulin sensitivity through Il-10 signaling (77, 78). Thus, mtDNA CN levels can potentially determine the metabolic signature of M1 vs M2 ATMs. Increased oxidative stress through elevated production of reactive oxygen species in diabetes can also impact on mitochondrial function, morphology and mtDNA replication (79). Although strong conclusions on exact causality cannot be made, these studies shed light on the potential mechanisms that may underpin the observed associations between mtDNA CN and metabolic syndrome.

This analysis considers mtDNA CN as an exposure variable driving metabolic outcomes, an approach adopted by several researchers. It is acknowledged that no causal direction can be robustly inferred from cross-sectional studies, and that reverse causation is a possibility. Fazzini et al. adopt a mediation analysis approach to show that a major proportion of the effect of mtDNA CN on T2DM was accounted for by obesity parameters (80). Insulin resistant states are accompanied by defects in mitochondrial function and structure, yet it is unclear whether these changes are mechanistically primary or secondary.

Our findings lend support for an impairment of mitochondrial function (as ascertained by reduced mtDNA CN) even when obesity is separated from its usual metabolic consequences. These findings are consistent across different definitions of metabolic health and suggest the absence of a healthy pattern of weight gain. This study is also strengthened by the use of a well-phenotyped representative cohort of middle-aged adults within a narrow age range. We used the same DNA extraction method in all participants. This is relevant as it has been demonstrated that DNA isolation methods influence mtDNA content measurement (81). In addition, this analysis corrected for differences in both amplification efficiency and blood cell type composition. Importantly, the abundant quantities of platelet mitochondria can artificially skew mtDNA CN, and correcting for cell composition is thus essential for interpretation (82, 83). All measurements were performed by one individual in a single laboratory, with random allocation of participants to minimize batch effects. To further minimize possible pre-analytic effects, care was taken to harmonize phlebotomy, sample transport and storage conditions.

This investigation carries several limitations. Primarily, its cross-sectional design limits evaluation of the interaction between mtDNA CN and body composition phenotypes along the developmental trajectory to cardiovascular disease endpoints. Peripheral blood leukocytes are a heterogenous cell population and this can dilute biological effect sizes. Leukocyte relative mtDNA CN estimates may not be directly extrapolated to more physiologically relevant tissues such as myocytes, hepatocytes, and adipocytes. This investigation centered only on mtDNA CN and did not focus on additional elements that could impact on mitochondrial bioenergetics and disease, such as mtDNA haplotypes and sequence variation (84, 85). The environmental and lifestyle determinants of mtDNA CN are poorly understood, with several factors being implicated (70). Importantly, statins have pleiotropic immunomodulatory effects and can negatively affect mitochondrial function and mtDNA CN (86). This study did not incorporate data on proinflammatory cytokines, diet, imaging-based assessment of visceral adiposity or cardiorespiratory fitness. We defined obesity by BMI thresholds which could have misclassified individuals with short stature or muscular build. No uniform definition of metabolic health exists in the scientific literature (87). Crucially, MHO is considered a transient phenotype having a high prevalence in premenopausal women, which can shift to a metabolically-unhealthy phenotype during the natural course of obesity and in response to obesity management (88, 89). The limited number of metabolically unhealthy normal weight subjects and unequal gender representation restricts interpretation of our findings. Unlike absolute measurements derived from digital PCR, qPCR generates relative measures of mtDNA CN that constraints comparison across studies.

## Conclusion

In summary, our findings reinforce the association between reduced leukocyte mtDNA copy number, obesity, and metabolic syndrome. While strong inferences on direction of causality cannot be made, this study highlights how obese individuals have a reduced mtDNA CN irrespective of healthy vs unhealthy designation. The distinction between the two may thus not be directly explained by pathophysiological changes at the level of the mitochondrion. The role of mtDNA CN in patient stratification requires further evaluation, and future efforts should strive to standardize the definition of metabolic health, explore causes of variation in mtDNA CN and evaluate the role of this biomarker in cardiometabolic risk classification. This process remains restricted by technical limitations in accurate and replicable quantification of mtDNA CN and by the lack of integration of mtDNA CN with multi-omic datasets that capture the genomic, proteomic and metabolomic landscape of metabolic disease.

## Data availability statement

The raw data supporting the conclusions of this article will be made available by the authors, without undue reservation.

## Ethics statement

The studies involving human participants were reviewed and approved by University Research Ethics Committe, UREC, University of Malta. The patients/participants provided their written informed consent to participate in this study.

## Author contributions

RA: sample collection. NP, RA: data analysis, drafting of manuscript. SF, NP, RA: study conception and design. SF, NP: project supervision. All authors contributed equally to the article and approved the submitted version.

## Funding

This research was supported by institutional funds from the University of Malta.

## Conflict of interest

The authors declare that the research was conducted in the absence of any commercial or financial relationships that could be construed as a potential conflict of interest.

## Publisher’s note

All claims expressed in this article are solely those of the authors and do not necessarily represent those of their affiliated organizations, or those of the publisher, the editors and the reviewers. Any product that may be evaluated in this article, or claim that may be made by its manufacturer, is not guaranteed or endorsed by the publisher.
