# Supplementary material for: Reduced leukocyte mitochondrial copy number in metabolic syndrome and metabolically healthy obesity
Source: Front Endocrinol (Lausanne). 2022 Jul 25;13:886957. doi: 10.3389/fendo.2022.886957 (PMC9357898; doi:10.3389/fendo.2022.886957)
Supplement: Supplementary file 1 [file DataSheet_1.docx]

Supplementary Material

## Supplementary Figure 1


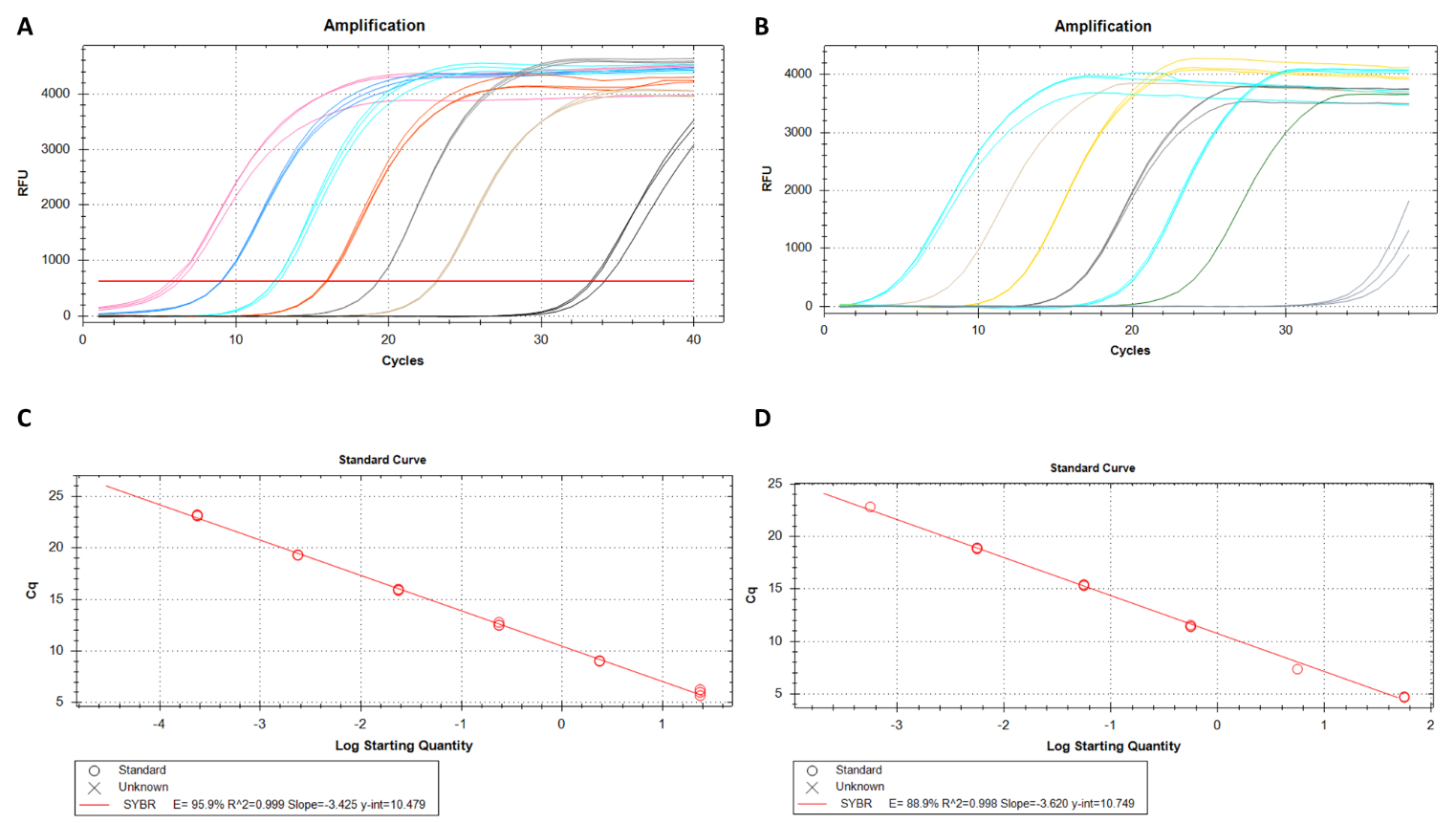


**Supplementary Figure 1. Representative amplification plots for *MT-CYTB* (A) and *HBB* (B)*.* Standard curves were constructed using six 10-fold serial dilutions of PCR products, and each standard dilution was amplified by real-time quantitative PCR using the *HBB* and *MT-CYTB* primer sets. A no-template control is included. Plots (C) and (D) show standard curves for *MT-CYTB* and *HBB* respectively, represented as a semi-log plot of Ct against starting concentration. The Ct values of unknown samples fell within the linear range.**

## Supplementary Figure 2


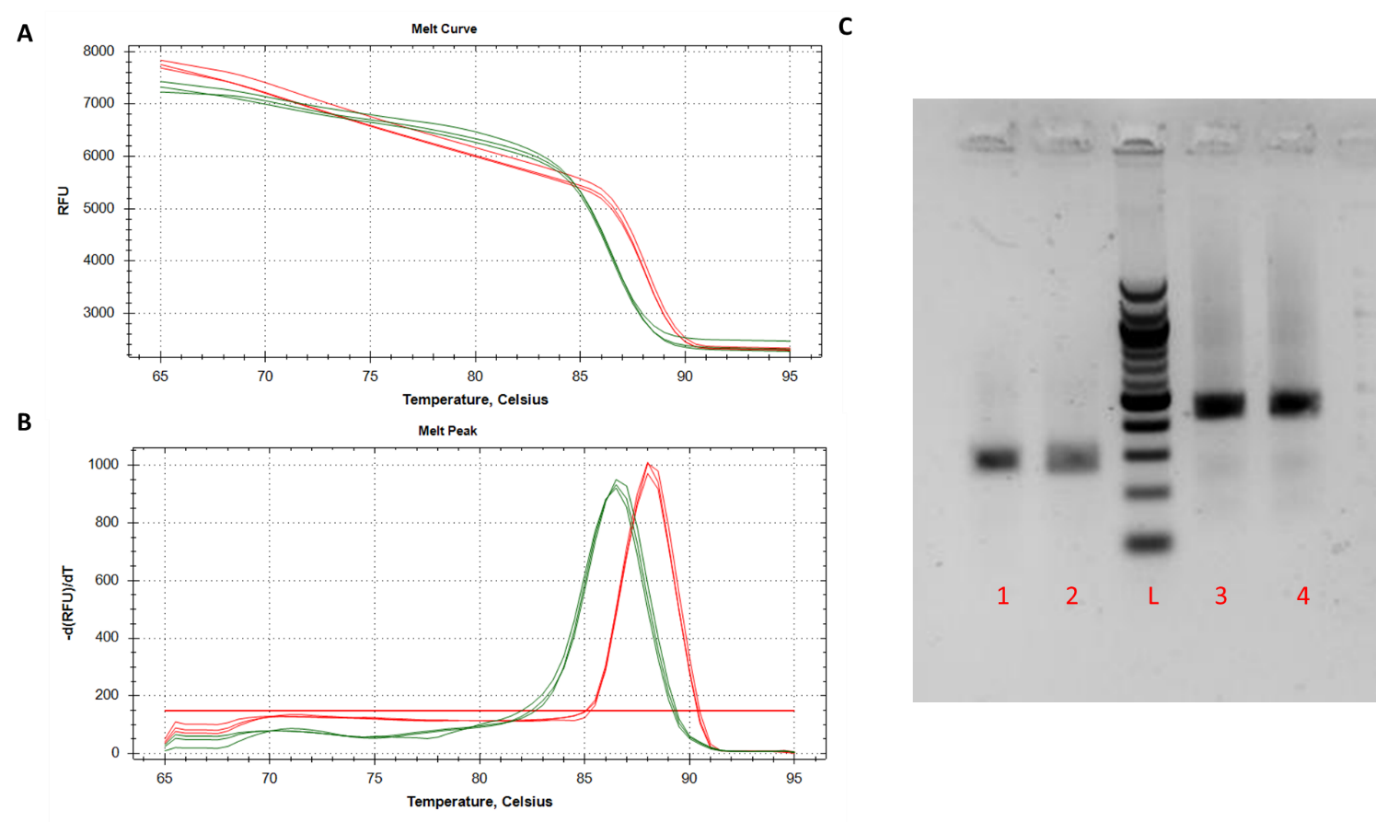


**Supplementary Figure 2. Melt curve (A) and melt peak (B) analysis of *MT-CYTB* (green) and *HBB* (red) triplicate amplicons. The dissociation curve charts the reduction in fluorescence observed during temperature ramping. The melt peak analysis is a plot of the negative first derivative of the dissociation curve and shows a characteristic peak for each product (the derivative is the negative of the rate of change in fluorescence as a fraction of temperature). C shows 2% agarose gel electrophoresis of PCR amplicons. Lanes 1 and 2 show the 268 bp *HBB* amplicon, and lanes 3 and 4 the 434 bp *MT-CYTB* amplicon. Lane L shows a 100bp DNA ladder (Solis Biodyne, Estonia).**

## Supplementary Figure 3


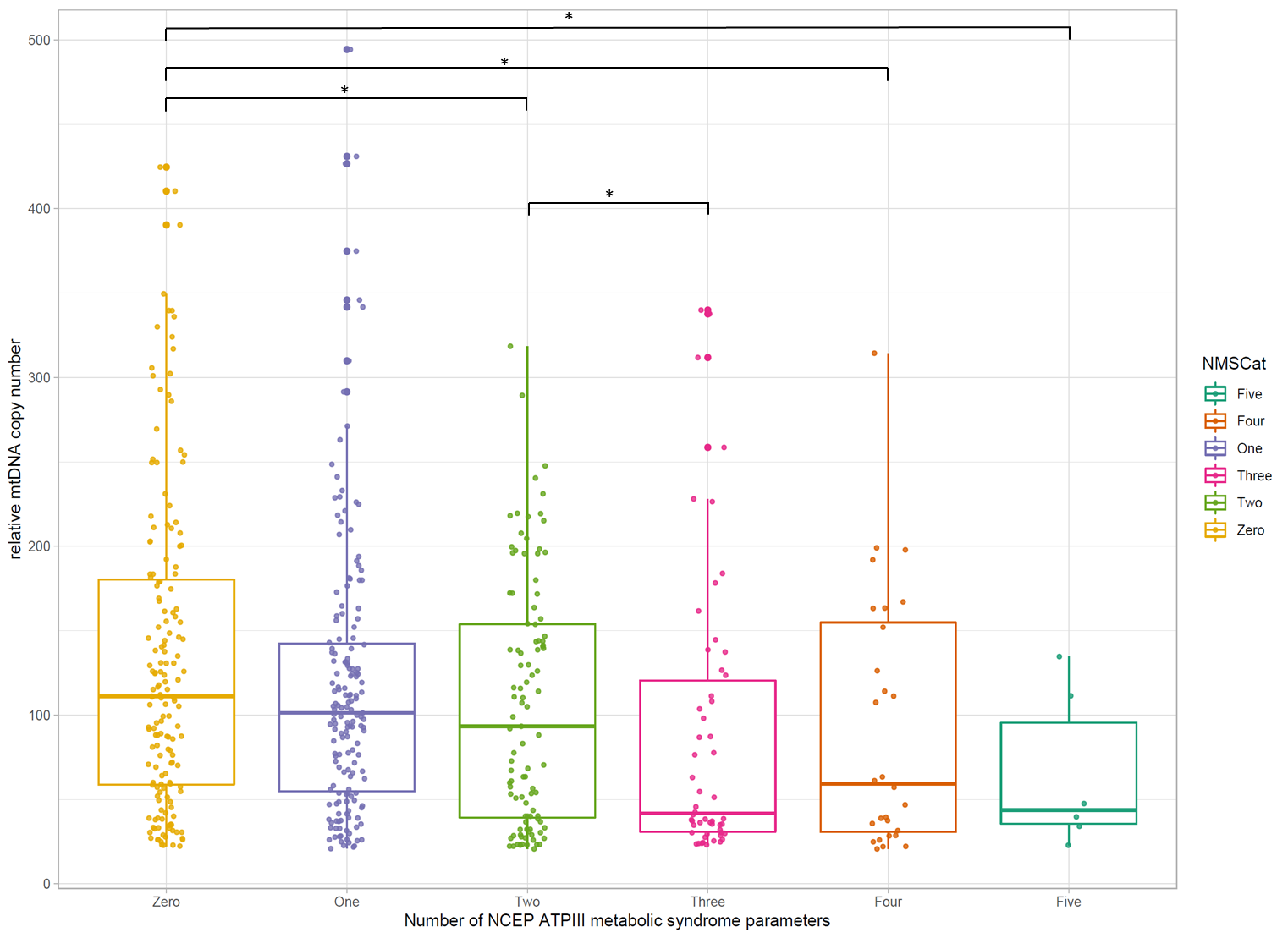


**Supplementary Figure 3. Box plot showing relative mtDNA copy number against number of NCEP-ATP III components of the metabolic syndrome. A significant reduction in mtDNA copy number with increasing metabolic syndrome components was observed (Independent samples Kruskal-Wallis ANOVA, p < 0.001). Pairwise comparison using Dunn’s post-hoc tests revealed significant differences between individual categories, indicated by * symbol.**

**Supplementary Table 1**

| **Variable** | **PC1** | **PC2** | **PC3** |
| --- | --- | --- | --- |
| **Waist circumference (cm)** | **0.906** | 0.069 | 0.189 |
| **Fasting plasma glucose (mmol/l)** | -0.015 | 0.045 | **0.833** |
| **HDL-C (mmol/l)** | -0.564 | -0.146 | -0.384 |
| **Triglycerides (mmol/l)** | 0.349 | 0.182 | **0.611** |
| **Body Mass Index (kg/m^2^)** | **0.895** | 0.054 | 0.1 |
| **Systolic BP (mmHg)** | 0.093 | **0.806** | 0.037 |
| **Diastolic BP (mmHg)** | 0.09 | **0.801** | 0.042 |
| **HOMA-IR** | 0.258 | -0.018 | **0.808** |
| **hs CRP (mg/l)** | 0.222 | -0.231 | 0.325 |

**Supplementary Table 1. Rotated component matrix derived from varimax rotation with Kaiser normalisation. Rotation reduces the number factors on which the variables under investigation have high loadings. The loadings represent correlations between each factor and the PC. PC1 is characterised by BMI and WC, PC3 is characterised by FPG and HOMA-IR and PC2 by systolic and diastolic blood pressure.**
